# Supplementary material for: Identification and functional analysis of non-coding regulatory small RNA FenSr3 in Bacillus amyloliquefaciens LPB-18
Source: PeerJ. 2023 May 15;11:e15236. doi: 10.7717/peerj.15236 (PMC10194069; doi:10.7717/peerj.15236)
Supplement: Supplemental Information 4 [file peerj-11-15236-s004.zip › KO/CK-vs-T1_map/map05210.html]

KEGG PATHWAY: Colorectal cancer - Reference pathway


|  |  |
| --- | --- |
| **Colorectal cancer - Reference pathway** |  |

[
Pathway menu
| Organism menu
| Pathway entry
| Show description
| User data mapping
]

Colorectal cancer (CRC) is the second largest cause of cancer-related deaths in Western countries. CRC arises from the colorectal epithelium as a result of the accumulation of genetic alterations in defined oncogenes and tumour suppressor genes (TSG). Two major mechanisms of genomic instability have been identified in sporadic CRC progression. The first, known as chromosomal instability (CIN), results from a series of genetic changes that involve the activation of oncogenes such as K-ras and inactivation of TSG such as p53, DCC/Smad4, and APC. The second, known as microsatellite instability (MSI), results from inactivation of the DNA mismatch repair genes MLH1 and/or MSH2 by hypermethylation of their promoter, and secondary mutation of genes with coding microsatellites, such as transforming growth factor receptor II (TGF-RII) and BAX. Hereditary syndromes have germline mutations in specific genes (mutation in the tumour suppressor gene APC on chromosome 5q in FAP, mutated DNA mismatch repair genes in HNPCC).

|  |  |  |
| --- | --- | --- |
| Reference pathway | 184% 150% 122% 100% 82% 67% 55% | 图片下载 |
